# The *PRMT1* gene expression pattern in colon cancer

**DOI:** 10.1038/sj.bjc.6604807

**Published:** 2008-12-09

**Authors:** K Mathioudaki, A Papadokostopoulou, A Scorilas, D Xynopoulos, N Agnanti, M Talieri

**Affiliations:** 1 Department of Cellular Physiology, ‘G Papanicolaou’ Research Center of Oncology, ‘Saint Savvas’ Hospital, 171 Alexandras Avenue, Athens 11522, Greece; 2 Department of Gastroenterology, ‘Saint Savvas’ Hospital, 171 Alexandras Avenue, Athens 11522, Greece; 3 Department of Biochemistry and Molecular Biology, Faculty of Biology, University of Athens, Panepistimioupoli, Athens 15711, Greece; 4 Department of Pathology, School of Medicine, University of Ioannina, Ioannina 45110, Greece

**Keywords:** protein arginine methyltransferase, PRMT1, colon cancer, prognosis

## Abstract

The methylation of arginine has been implicated in many cellular processes, such as regulation of transcription, mRNA splicing, RNA metabolism and transport. The enzymes responsible for this modification are the protein arginine methyltransferases. The most abundant methyltransferase in human cells is protein arginine methyltransferase 1. Methylation processes appear to interfere in the emergence of several diseases, including cancer. During our study, we examined the expression pattern of protein arginine methyltransferase 1 gene in colon cancer patients. The emerging results showed that the expression of one of the gene variants is associated with statistical significant probability to clinical and histological parameters, such as nodal status and stage. This is a first attempt to acquire an insight on the possible relation of the expression pattern of protein arginine methyltransferase 1 and colon cancer progression.

Colon cancer is one of the most dominant types of cancer in Western industrialised countries. There are many hereditary syndromes that significantly increase the possibility of colon cancer occurrence, such as familial adenomatous polyposis coli and hereditary non-polyposis coli. Almost 40% of colorectal cancers are diagnosed with localised disease, which have approximately a 90% 5-year survival rate. However, the prognosis worsens with advancing stage, and only 5% of patients diagnosed with distant metastases survive 5 years (Duffy *et al*, 2003). The need for biomarkers indicative of cancer status is becoming more and more evident. Biological markers represent very strong tools for monitoring the progression of cancer and estimating the effectiveness and safety of new therapeutic agents (Cho, 2007).

Protein function is often dependent on modifications that occur on their amino-acid residues. Protein methylation is one such modification that involves the transport of methyl groups from *S*-adenosylmethionine (AdoMet) to target molecules on the protein substrates. The proteins can be methylated on several residues, such as arginine residues among others (Aletta *et al*, 1998). The methylation of arginine residues on proteins is involved in a number of different cellular processes, such as regulation of transcription, RNA metabolism and DNA damage repair (Bedford and Richard, 2005). The enzymes responsible for this modification are called protein arginine methyltransferases (PRMTs). The PRMTs comprise a family of nine protein members so far (Lin *et al*, 1996; Scott *et al*, 1998; Tang *et al*, 1998; Chen *et al*, 1999; Pollack *et al*, 1999; Frankel *et al*, 2002; Miranda *et al*, 2004; Lee *et al*, 2005b; Cook *et al*, 2006) and can be classified into three distinct classes. Type I enzymes catalyse the formation of N^G^-monomethylarginine and asymmetric N^G^, N^G^-dimethylarginine residues. Type II enzymes catalyse the formation of N^G^-monomethylarginine and symmetric N^G^, N′^G^-dimethylarginine residues. Type III enzymes catalyse monomethylation of the internal guanidine nitrogen atom to form *ω*-N^G^-monomethylarginine (Zobel-Thropp *et al*, 1998).

The predominant mammalian protein arginine methyltransferase is PRMT1 (Tang *et al*, 1998; Frankel *et al*, 2002). Protein arginine methyltransferase 1 is a multifunctional protein, implicated in diverse biological processes, such as DNA repair, signal transduction, protein trafficking and RNA processing (McBride and Silver, 2001; Boisvert *et al*, 2003; Bedford and Richard, 2005; Lee *et al*, 2005b). Protein arginine methyltransferase 1 methylates a number of hnRNP molecules, playing a role in the shuttling of these proteins between the cytoplasm and the nucleus (Herrmann *et al*, 2004), and histone H4 at arginine 3 (Wang *et al*, 2001), a modification that functions as a transcription activation mark. The central role that PRMT1 plays as a regulator of protein function is shown by the disruption of this enzyme in mice. The *PRMT1*-knockout mice die shortly after implantation (Pawlak *et al*, 2000). Protein arginine methyltransferase 1 prefers to methylate arginine residues in glycine and arginine rich regions that are often found in RGG repeats of proteins, such as fibrillarin and nucleolin (Najbauer *et al*, 1993; Lee *et al*, 2005a). The *PRMT1* gene is found on chromosome 19q13.3, in close proximity to the genes *RRAS* and *IRF3*. Genomic organisation, physical mapping and expression analysis of *PRMT1* gene has already been reported by Scorilas *et al* (2000).

Primary immunocytochemical localisation studies on RAT1 cells suggested that PRMT1 is predominantly nuclear (Tang *et al*, 1998). In later projects, there has been evidence of the presence of PRMT1 in the cytoplasm, suggesting the continuous translocation of the protein between the nucleus and the cytoplasm (Araya *et al*, 2005). Furthermore, according to Herrmann *et al*, 2005, PRMT1 is a highly dynamic enzyme with variable subcellular localisation and mobility.

Methylation processes have been implicated in the emergence and progression of several diseases, including cancer (Shin *et al*, 1993; Li *et al*, 1998; Gu *et al*, 1999). In this study, the expression of *PRMT1* mRNA was determined, for the first time, by semiquantitative RT–PCR in 120 colon cancer tissues (for 60 of which paired normal colon mucosa was also examined), 14 adenomas and 24 biopsies of inflamed colon mucosa. The results were associated with other clinical and pathological parameters. In addition, a defined number of normal and colon cancer samples were examined by immunohistochemistry using in-house-produced polyclonal antibody (anti-PRMT1 IgG) to determine the localisation of the protein.

## Materials and methods

### Study group

The tumour specimens of our study were acquired from 120 patients who underwent surgery for primary colon cancer at the ‘Saint Savvas’ Oncologic Hospital of Athens. For 60 out of the 120 patients, paired normal colon mucosa was also examined. Apart from these, an additional group of 14 adenomas and 24 biopsies of inflamed colon mucosa were included in the study. Informed consent was obtained from all patients. The samples were followed by an updated database containing clinical and histological information about the tumour (tumour size, grade, stage, etc.) that was used for the statistical analysis of the results. Patients' age ranged from 31 to 92 years, with a mean of 67.45±1.08 years. Follow-up information, which was available for 112 patients, showed that 29 (25.9%) of them had relapsed and 47 (42%) had died. The staging was performed according to the TNM staging system, as introduced by the American Joint Committee on Cancer.

### RNA extraction and semiquantitative RT–PCR

Colon tissues were collected on surgery and kept in liquid nitrogen. The tissue samples were pulverised (Polytron, Kinematica AG, Lucerne, Switzerland) and total RNA was extracted using TRIzol (Invitrogen, Carlsbad, CA, USA), according to the manufacturer's instructions. Following spectrophotometry determined the purity and concentration of the RNA. The total RNA was reverse-transcribed by RT–PCR using the Thermoscript^-^RT (Invitrogen). The integrity of the produced cDNA was examined by amplification of *β-actin* gene (housekeeping gene). PCR was performed in a 20 *μ*l reaction mixture containing 0.6 *μ*l of cDNA, 2 U of Platinum *Taq* DNA polymerase (Invitrogen), 2 *μ*l of 10 × PCR buffer (200 mM Tris–HCl (pH 8.4), 500 mM KCl), 0.8 *μ*l of 50 mM MgCl_2_, 0.4 *μ*l of 10 mM dNTPs mix (Invitrogen) and 0.4 *μ*l of each gene specific primer (0.1 *μ*g *μ*l^−1^) (Table 1). The amplification protocol consisted of an initial incubation at 95°C for 15 min, followed by 35 cycles of 95°C for 30 s (denaturing step), 62°C for 1 min (annealing step), 72°C for 1 min (extension step) and a final extension step of 72°C for 10 min. PCRs were performed on the PTC-200 thermal cycler (MJ Research Inc., Waltham, MA, USA).

**Figure 1:**
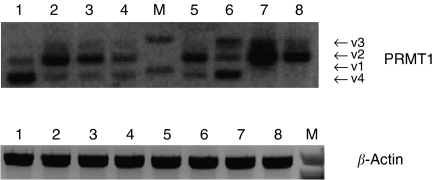
Expression of *PRMT1* in normal and cancerous colon tissue.

The *PRMT1* gene was amplified using a specific set of primers (Table 1). PCR was performed in a 20 *μ*l reaction mixture containing 0.8 *μ*l of cDNA, 2 U of Platinum *Taq* DNA polymerase (Invitrogen), 2 *μ*l of 10 × PCR buffer (200 mM Tris–HCl (pH 8.4), 500 mM KCl), 0.8 *μ*l of 50 mM MgCl_2_, 0.4 *μ*l of 10 mM dNTPs mix (Invitrogen) and 0.4 *μ*l of each gene specific primer (0.1 *μ*g *μ*l^−1^). The amplification protocol consisted of an initial incubation at 95°C for 15 min, followed by 40 cycles of 95°C for 30 s (denaturing step), 64.5°C for 1 min (annealing step), 72°C for 1 min (extension step) and a final extension step of 72°C for 10 min. PCRs were performed on the PTC-200 thermal cycler (MJ Research Inc.). The identity of the products was verified by sequencing, with an automated DNA sequencer. Equal amounts of PCR products for *β-actin* and *PRMT1* ran on 1.5% agarose gel, and visualisation was based on ethidium bromide staining (Figure 1). Densitometric measurements of band intensities using the Gel Logic 100 Imaging System and the 1D Image Analysis Software, version 3.6 (Eastman Kodak Company, Rochester, NY, USA) were used to calculate the ratio of *PRMT1/β-actin*. On the basis of this ratio, the expression of *PRMT1* was characterised as high or low, after comparison with the mean ratio of non-cancer samples. Expression analysis was performed twice for each sample.

### Statistical analysis

The expression of the *PRMT1* splice variants was classified as high or low and was correlated with the clinical and histopathological features of the patients. The associations between these variables and *PRMT1* status of each variant were analysed using the *χ*^2^ test or the Fisher's Exact test, where appropriate. Regression analysis was performed using the Cox proportional hazard regression model (Cox, 1972) at both univariate and multivariate levels. The multivariate models were adjusted with TNM stage, patients' age and tumour grade. Survival analyses were carried out by constructing Kaplan–Meier disease-free survival (DFS) curves and overall survival (OS) curves (Kaplan and Meier, 1958) for all variants of *PRMT1*. The differences between the curves were calculated by the log-rank test.

### Production of antibodies

Rabbit polyclonal antibodies against PRMT1 were raised in New England white rabbits in our lab. The peptide used as an antigen for the antibody production had the amino-acid sequence FGTIGMRPNAKNNRDLDFTI and it was synthesised by Invitrogen. The produced antibody was purified by affinity chromatography using A-Sepharose protein beads kindly provided to our lab by Dr Pigi Lymberi (Department of Biochemistry, Immunology Lab, Institut Pasteur Hellenique, Athens, Greece).

### Immunohistochemistry

Formalin-fixed paraffin-embedded colon tissues from selected patients expressing PRMT1 were used for the study. Tissue sections (5 *μ*m in thickness) were microwave-treated for 15 min in 1 mM sodium citrate buffer solution (pH=6.0) for the unmasking of the antigen, after deparaffinisation and rehydration with xylene and a series of graded ethanols (100, 90 and 70%). Endogenous peroxidase activity was blocked with 3% H_2_O_2_ for 5 min, followed by 2 min washes under flowing normal tap water. The tissue sections were incubated with 200 *μ*l of primary polyclonal antibody against PRMT1 (anti-PRMT1 IgG), produced in our lab, diluted 1 : 1500, for 1.5 h at room temperature.

Sections were washed for 5 min with phosphate-buffered saline (PBS) solution (pH=7.4) and incubated for 40 min with 200 *μ*l reagent HRP Rabbit/Mouse (ENV) (Dako REAL™ EnVision™ Detection System Peroxidase/DAB+, Rabbit/Mouse, Glostrup, Denmark), containing secondary goat anti-rabbit IgG. Sections were washed again for 5 min with PBS and incubated with 150 *μ*l of reagent DAB+ chromogen (50 × ) diluted 1 : 50 in substrate buffer (Dako REAL™ EnVision™ Detection System Peroxidase/DAB+, Rabbit/Mouse) for 10 min. Sections were washed for 5 min with PBS, counterstained for 1 min with haematoxylin (Hemacolor, Merck KGaA, Darmstadt, Germany), dehydrated, cleared and mounted with DePex (BDH, Limited Poole, Dorset, UK). Negative controls had the primary antibody omitted and replaced by PBS. Two pathologists who were unaware of patient history evaluated all samples.

## Results

### Identification of a new PRMT1 splice variant

The *PRMT1* gene was known to give rise to three distinct isoforms of the same enzyme (Scott *et al*, 1998; Scorilas *et al*, 2000), due to alternative splicing. The sequence of the splice variants that produce each isoform have been reported by Scorilas *et al* (2000), and the variants have been characterised as v1, v2 and v3, v1 being the shortest one. During the course of this study, the emergence of an additional gene product came to our attention. After isolation and sequencing, it appeared to be a new splice variant, 48 bp smaller than v1 that was accessed to GenBank by our lab (Accession Number AY775289).

### *PRMT1* status and relation to clinical and histopathological variables

The expression analysis of *PRMT1* in colon cancer patients showed that only the *PRMT1*-v1 status and in some cases *PRMT1*-v2 status effectively associate with clinical or histological parameters, of all four variants expressed in colon tissue, due to alternative splicing.

Table 2 shows the distribution of numerical variables in the study group. Table 3 presents the expression pattern of variant *PRMT1*-v1 in the different colon tissues. We have used as a cutoff value the mean of the background values +2 s.d. *P-*value was calculated using *χ*^2^ test and shows that high expression of *PRMT1*-v1 is associated with statistical significant probability to the type of tissue (*P*<0.001). The statistical analysis of the *PRMT1*-v1 status in relation to nodal status, TNM stage and tumour grade was performed using the parametric Fisher's Exact test and the *χ*^2^ test (Table 4). High expression of *PRMT1*-v1 seems to associate in a statistically significant manner with highly malignant tumours (*P*=0.005), tumours of advanced TNM stage (*P*=0.001) and tumours with positive nodal status with a low statistical power (*P*=0.042). Statistically significant relationships between tumour size and *PRMT1* expression levels was not observed while using the Mann–Whitney non-parametric test.

### Survival analysis

**Figure 2:**
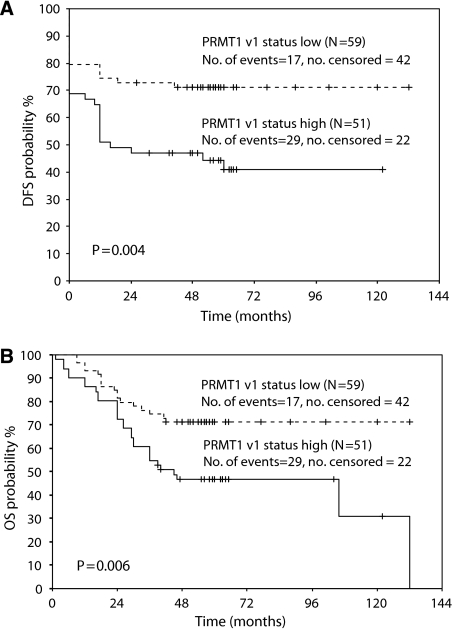
Prognostic value of PRMT1-v1 expression in colon cancer patients (*P*-value was calculated by the log-rank test. The small vertical lines indicate the censoreds. Censored cases refer to the number of patients that have not reached the terminal event during our study).

**Figure 3:**
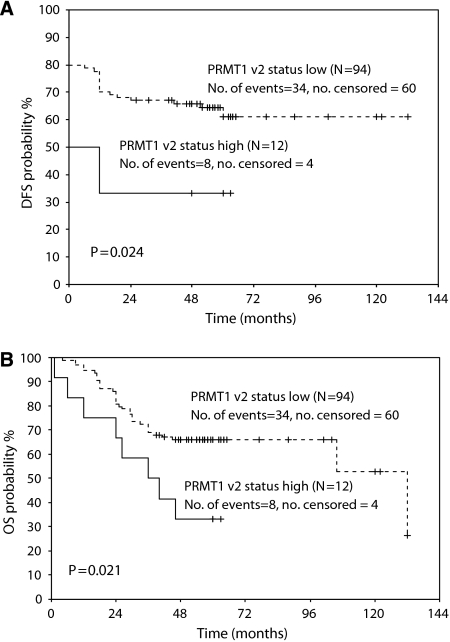
Prognostic value of PRMT1-v2 expression in colon cancer patients (*P*-value was calculated by the log-rank test. The small vertical lines indicate the censoreds. Censored cases refer to the number of patients that have not reached the terminal event during our study).

The associations between *PRMT1* gene expression and DFS as well as OS, in both univariate and multivariate analysis, are presented on Table 5. According to Cox univariate analysis, high expression of *PRMT1*-v1 is significantly associated with the OS (HR=2.24, *P*=0.008) and, to a smaller extent, with DFS (HR=3.81, *P*=0.011), whereas high expression of *PRMT1*-v2 seems to associate only with OS (HR=2.41, *P*=0.026). On the other hand, multivariate analysis shows no significant relationship between *PRMT1* expression and DFS or OS. Survival curves created according to the Kaplan–Meier method for DFS and OS showed that low expression of *PRMT1*-v1 relates to longer DFS and OS (*P*=0.004 and *P*=0.006, respectively) (Figure 2). Additionally, low expression of *PRMT1*-v2 seems to associate with longer DFS and OS in a similar manner (*P*=0.024 and *P*=0.021, respectively) (Figure 3).

### Localisation of the PRMT1 protein

**Figure 4:**
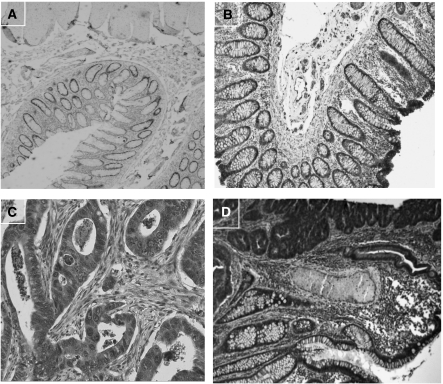
Immunohistochemical localisation of PRMT1 protein in colon tissue. (**A**) Normal colon, negative control (primary antibody omitted) ( × 100), (**B**) normal colon tissue ( × 125), (**C**) cancerous colon tissue ( × 150) and (**D**) cancerous colon tissue ( × 100).

Selected cancer and normal colon samples were stained for PRMT1 (Figure 4). The primary antibody against PRMT1 was raised in our lab and its binding ability to the substrate was checked on western blotting. In both normal and cancer colon tissues staining is very intense on the cytoplasm and only in rare cases the cell nucleus appeared stained.

## Discussion

Protein arginine methylation is a major post-translational modification that seems to regulate a variety of cellular processes, such as signal transduction, RNA metabolism, chromatin structure, RNA and protein trafficking. The enzymes responsible for this type of modification are the PRMTs. Protein arginine methyltransferase 1 represents the prevalent protein methyltransferase in human cells. The major targets of PRMT1 include components of the hnRNPs (Nichols *et al*, 2000), histone H4 (Wang *et al*, 2001) and peroxisome proliferator-activated receptor *γ* coactivator 1*α* (Teyssier *et al*, 2005).

It is known for a long time now that alterations in the expression pattern of certain genes are strongly correlated with cancer incidence. Most of these genes are involved in developmental processes and cell cycle-regulatory events, such as *MLH1* and *MLH2*, *TP53*, *LKB1*, as far as colon cancer is concerned. Although the physiological role of PRMT1 is not fully elucidated yet, several hypotheses have been made. Among others, a role in cancer has been suggested for this enzyme. In this study, the possible role of *PRMT1* as a new biomarker for colon cancer was examined. The tissue samples used for the study were not selected for proximal or distal splenic flexure, as, so far, evidence as to the existence of two sides in colorectal cancer from different studies are still quite contradictory (Iacopetta, 2002).

Statistical analysis of the results, concerning semiquantitative measurements of *PRMT1* expression, showed association between the expression of *PRMT1*-v1 splice variant and clinical and histological data of the tumours. First of all, the expression of v1 variant seems to increase following the progression from normal tissue to adenoma and finally cancer. The levels of v1 isoform are significantly higher in colon cancer tissue compared with normal tissue. In accordance with the above findings, high expression of *PRMT1*-v1 seems to be indicative of the disease progression and aggressiveness. It seems that the higher the malignancy grade, the greater the expression of the variant. Additionally, both advanced TNM stage and presence of positive lymph nodes are linked in a statistically significant manner to high v1 variant expression. Survival analysis was performed with both Cox proportional hazard regression model and Kaplan–Meier method. The univariate Cox analysis showed a profound relation of high *PRMT1*-v1 expression and both DFS and OS of patients. Patients expressing strongly the v1 variant show higher probability to relapse or die, compared with patients with low expression. In line with univariate Cox regression analysis, Kaplan–Meier survival plots also indicate a low survival probability for the individuals that express highly the v1 variant.

As far as the other three isoforms are concerned, the association between their expression patterns and other parameters is not very strong. In the case of v2, survival analysis gives low survival probabilities and higher chance of relapse for patients highly expressing the v2 variant. But these associations are much weaker than those regarding the expression of *PRMT1*-v1. Similarly, the presence of *PRMT1*-v1 seems to be associated with clinical and pathological features and poor prognosis in breast cancer patients (our unpublished data).

Approximately, 10–30% of alternatively spliced human genes have tissue-specific variants (Xu *et al*, 2002), whereas 316 genes have been shown to have cancer-specific variants (Xu and Lee, 2003). Production of alternative transcripts may be a consequence of disease such as cancer (Mercatante and Kole, 2000). These findings seem to be in accordance with the presence of *PRMT1*-v4 splice variant in colon tissue (normal and cancerous) but not in breast tissue (our unpublished data).

The product of *PRMT1* gene is still under intense study. There are many things to be defined and clarified. One of them is the behaviour of the protein in the cell. There is conflicting evidence about the localisation site of the protein (Tang *et al*, 1998; Araya *et al*, 2005). It is known that in different cell lines the proteins in need of methylation can vary both in quantity and quality. As a result, methyltransferases are expected to function either in the nucleus or the cytoplasm, depending on the localisation of their substrates. Results from the immunohistochemical experiments performed in our lab, in normal and cancer colon tissues, indicated that the protein resides mainly in the cytoplasm. Its presence in the nucleus is rare. The same pattern was noticed in breast tissue, which is also epithelial (our unpublished data). It is quite strange for an enzyme that methylates arginine residues mostly on nuclear proteins to be found to such an extent in the cytoplasm. This could probably be attributed to the fact that methylation occurs during or shortly after translation, so the enzyme needs to reside in the cytoplasm. The mechanism used by the protein to exit the nucleus at one time and still be able to return is yet unknown. Experiments by Herrmann *et al* (2005) indicate that PRMT1 has the ability to move between nucleus and cytoplasm, probably piggybacked on unmethylated substrates that carry them through the nuclear pore (Herrmann *et al*, 2005).

Data from this study suggest that *PRMT1* gene variant v1 expression may be used as a marker of unfavourable prognosis for colon cancer patients. Studies on the physiological function of PRMT1 in normal colon will shed more light into the role of this enzyme in cancer as well as other diseases. The *PRMT1* gene combined with other markers could be proven useful for physicians. Nevertheless, more extensive study is needed to establish a role for *PRMT1* as a potential biological prognostic marker.

## Figures and Tables

**Table 1:** Gene-specific primers used for the amplification of *β-actin* and *PRMT1* cDNA

**Gene**	**Primer sequence**	**Product size (bp)**
*β-Actin*	Forward 5′-ATCTCGCACCACACCTTCTA-3′	372
	Reverse 5′-CGTCATACTCCTGCTTGCTG-3′	
PRMT1	Forward 5′-GAGGCCGCGAACTGCATCAT-3′	283, 331, 385, 502
	Reverse 5′-TGGCTTTGACGATCTTCACC-3′	

**Table 2:** Distribution of numerical values

**Variable**	**Mean+s.e.**	**Range**	**Percentiles**				
			**10**	**25**	**50**	**75**	**90**
			**(Median)**				
Age (years)	67.5±1.1	31–92	51.6	60.0	70.0	76.0	81.0
Tumour size (cm)	4.48±0.17	1.80–12.0	2.57	3.50	4.20	5.05	6.50
DFS (months)	37.3±2.8	0.0–132.0	0.0	0.0	48.0	58.0	64.0
OS (months)	46.7±2.4	1.0–132.0	12.0	26.0	48.0	58.7	65.0

**Table 3:** Expression status of *PRMT1* variant v1 in colon tissues

**Tissue**		**No. of patients (%)**		
	**Total**	**PRMT1-v1 status low^a^**	**PRMT1-v1 status high^a^**	***P*-value^b^**
Non-cancer	83	72 (86.7)	11 (13.3)	
Adenomas	14	11 (78.6)	3 (21.4)	**<0.001**
Cancer	120	63 (52.5)	57 (47.5)	

a PRMT1/*β*-actin.

b *χ*^2^ test. Statistically significant values are in bold.

**Table 4:** Associations between status of PRMT1 variant v1 and other variables in colon cancer patients

		**No. of patients (%)**		
**Variable**	**Total**	**PRMT1-v1 status low^a^**	**PRMT1-v1 status high^a^**	***P*-value**
*Nodal status*				
Negative	40	27 (67.5)	13 (32.5)	**0.042^b^**
Positive	61	28 (45.9)	33 (54.1)	
				
*TNM stage*				
I	44	31 (70.5)	13 (29.5)	**0.001^b^**
II–III	44	15 (34.1)	29 (65.9)	
				
*Grade*				
I	9	6 (66.7)	3 (33.3)	
II	36	26 (72.2)	10 (27.8)	**0.005^c^**
III	46	17 (37.0)	29 (63.0)	

a PRMT1/*β*-actin.

b Fisher's exact test.

c *χ*^2^ test. Statistically significant values are in bold.

**Table 5:** Associations of PRMT1 variants v1 and v2 expression with DFS and OS

	**Disease-free survival**			**Overall survival**		
**Variable**	**HR^a^**	**95% CI^b^**	***P*-value**	**HR^a^**	**95% CI^b^**	***P*-value**
*Univariate analysis*						
*PRMT1-v1*						
Negative	1.00			1.00		
Positive	3.81	1.35–10.73	**0.011**	2.24	1.23–4.09	**0.008**
						
*PRMT1-v2*						
Negative	1.00			1.00		
Positive	1.66	0.38–7.32	0.49	2.41	1.11–5.22	**0.026**
						
*Multivariate analysis* c						
*PRMT1-v1*						
Negative	1.00			1.00		
Positive	1.69	0.54–5.32	0.37	0.84	0.366–1.94	0.69
						
*PRMT1-v2*						
Negative	1.00			1.00		
Positive	0.38	0.041–3.61	0.41	0.98	0.35–2.71	0.97

a Hazard ratio (HR) estimated from Cox proportional hazard regression model.

b Confidence interval of the estimated HR.

c The multivariate models were adjusted with TNM stage, patient age and tumour grade. Statistically significant values are in bold.
